# Apathy - where do we find it and how to treat

**DOI:** 10.1192/j.eurpsy.2022.2275

**Published:** 2022-09-01

**Authors:** R. Gomes, C. Santos, N. Descalço, F. Moutinho

**Affiliations:** Hospital Garcia de Orta, Psychiatry, Almada, Portugal

**Keywords:** Treatment, apathy, Neuropsychiatry

## Abstract

**Introduction:**

Although defined heterogeneously within the literature apathy is classified as a multidimensional deficit with emotional, behavioral, and cognitive domains in which there is a decrease in self-motivated/goal-directed activity. Recently conceptualized as a syndrome but lacking a universal screening tool.

**Objectives:**

Review current knowledge on apathy and its best therapeutic approach.

**Methods:**

Non-systematic review of literature through search on PubMed/MEDLINE following the terms “apathy”and“psychiatry”.

**Results:**

Apathy is amongst the most frequent symptoms of dementia and highly prevalent across different forms and stages of dementia, including mild cognitive impairment (MCI) as well as other neurodegenerative and psychiatric disorders such as Parkinson’s disease (PD), Schizophrenia, Depression and Brain Injury. Individuals with apathy have higher frequencies of cognitive impairment and are less likely to be compliant/respond to treatment for comorbid illnesses. Apathy reduces quality of life, increases mortality and leads to caregivers distress - often identified as the most burdensome symptom. Once treatment should favor dopaminergic neurotransmission, psychostimulants were considered. Methylphenidate showed encouraging results as well as dopamine agonists but both with limited evidence. Atypical antipsychotics(APs) seem beneficial compared to typical APs. Antidepressants did not improve symptoms and may even worsen them. Previously reported benefits of acetylcholinesterase inhibitors (AChEIs) were not replicated in recent studies except for rivastigmine in PD. Nonpharmacological interventions are also important.

**Conclusions:**

Apathy occurs frequently in a broad range of neuropsychiatric conditions and considering its impact on patients´ quality of life more studies are needed to find an efficient treatment. A consensus regarding definition and screening tools would allow a better approach.

**Disclosure:**

No significant relationships.

